# The influence of real estate brokers’ personalities, psychological empowerment, social capital, and knowledge sharing on their innovation performance: The moderating effect of moral hazard

**DOI:** 10.3389/fpsyg.2022.971339

**Published:** 2022-09-21

**Authors:** Hung-Chung Chang, Chun-Chang Lee, Wen-Chih Yeh, Yi-Lun Chang

**Affiliations:** ^1^Department of Business Administration, Chihlee University of Technology, New Taipei City, Taiwan; ^2^Department of Real Estate Management, National Pingtung University, Pingtung, Taiwan; ^3^Department of Real Estate Management, HungKuo Delin University of Technology, New Taipei City, Taiwan

**Keywords:** personality, psychological empowerment, social capital, knowledge sharing, innovation performance, moral hazard

## Abstract

This study proposed and examined a conceptual framework on the influence of real estate brokers’ personalities, psychological empowerment, social capital, and knowledge sharing on their innovation performance, and used moral hazard as a moderating variable. We used structural equation modeling (SEM) for data analysis and estimation. The participants were real estate brokers in Kaohsiung City. A total of 1,000 questionnaires were administered to 100 branch offices of real estate companies, 571 of which were later recovered from 80 branch offices. After removing 52 for being invalid, we were left with 519 valid questionnaires, indicating a 51.9% recovery rate. The empirical results suggest that the real estate brokers’ personalities and psychological empowerment positively and significantly influence social capital; personality and psychological empowerment also positively and significantly influence their innovation performance; and social capital significantly influences knowledge sharing, which in turn positively and significantly influences the brokers’ innovation performance. In environments with higher levels of moral hazard, the influence of social capital on the brokers’ knowledge sharing is significantly diminished.

## Introduction

A company’s innovation performance signifies its developmental capability and is also a key indicator in the service industry. Lee (2007) argued that, for the service industry, innovation is an important determinant of a company’s competitive advantage. Porter (1985) wrote that a company outputs its performance and gains its competitive advantage by improving its services to satisfy customer demands. In the highly competitive and service-centered real estate industry, the method with which brokers create new services is crucial for their future industry survival.

Innovation is the process of acquiring original and valuable new products, processes, or services by means of displaying, combining, or integrating knowledge (Luecke and Katz, 2003). Previous studies have revealed several factors covering the financial, service, management, and technical aspects that affect innovation performance in the real estate industry. These include the employees’ remuneration, business performance, and number of transactions; the organization’s financial status (Lee, 2004; Yu and Liu, 2004; Lee and You, 2007); talent development; stigmatized housing compensation services; and app-based property viewing systems (Yang, 2012).

Real estate falls within the service industry. The close interactions and relationships between industry players often involve knowledge sharing. Social capital is a means to study personal relations. In recent years, related studies have argued that social capital is based on the establishment of trust-based networks, and the status of the social networks within an organization affect the production of organizational knowledge (Nahapiet and Ghoshal, 1998; Thompson, 2018). This is because a wealth of both information and resources are exchanged and transferred through human-to-human interactions. In traditional organizational behavior studies, this data is observable, but difficult to measure (Lee, 2007). To acquire or transfer these resources and knowledge, companies must establish good relationships with the partners within their networks. Moreover, different external partners possess different resources and capabilities, thereby providing different contributions to a company’s innovation (Gemündena et al., 1996). Lesser (2000) suggested that social capital strengthens knowledge acquisition. Indeed, a company can improve its internal knowledge learning capacity by enhancing the trust between employees. Therefore, we can reasonably assume that social capital influences knowledge sharing, which in turn affects innovation performance. Zárraga and Manuel García-Falcón (2003) have previously stated that the multidirectional communication between organizational members is conducive to enhancing the organization’s innovation and innovation performance.

Additionally, personality-related factors, such as personality and psychological empowerment, are important to real estate brokers’ interpersonal relations or innovation performance. Personality is a stable and crucial component in life (Costa and McCrae, 1992). Franco and Prata (2018) mentioned that individuals who are extroverted, responsible, and open to experience positively influence their performance, whereas those who are easily nervous compromise their performance. Studies have demonstrated that extroverted individuals find it easy to attract the attention of others while maintaining interactive interpersonal relations, thereby strengthening their social capital. The aforementioned studies have indicated that personality influences an individual’s innovation performance and social capital (Ashton et al., 2002). Bowen and Lawler (1992) suggested that psychological empowerment not only enables employees to have authority, but also facilitates their sharing of information, knowledge, and rewards with other employees. Therefore, psychological empowerment entails autonomy and independence. Spreitzer (1995) wrote that psychological empowerment is an intrinsic task-oriented motivation that drives a person to take the initiative and consistently complete organizational goals by leveraging their evaluation and perception of a work task. Lawler (1990) suggested that enhanced psychological empowerment leads to higher innovation, performance, and productivity. Knight (2006) believed psychological empowerment to be closely related to innovation performance. Christens (2012) suggested that psychological empowerment can be theorized into affective, behavioral, and cognitive structural components. The process of psychological empowerment is expanded through, and reliant on, the development of interpersonal relations. The above studies thus suggest that psychological empowerment influences an individual’s innovation performance and social capital.

The information above suggests that social capital influences knowledge sharing, which in turn impacts innovation performance. In economies reliant on capital markets, informational asymmetry is likely to occur between buyers and sellers, which leads to the risk of moral hazard (Chen and Shiu, 2020). Because real estate transactions involve large cash flows, there are often bank loans and mortgages involved (Lin, 2008). These transactions and are also associated with a broker’s personal sales performance. Given the informational asymmetry in this market, brokers are easily incentivized by the promise of personal gains since they have the advantage of holding information. Consequently, the risk of moral hazard increases. While knowledge sharing is a voluntary behavior, it is innate human nature to conceal and not necessarily share information with others freely, so as to maintain one’s own competitive advantage (Weng and Tung, 2017). This behavior influences the effect of social capital on knowledge sharing, and is also easily confounded by moral hazard. This study explores whether the effect of social capital on knowledge sharing is confounded by moral hazard. Previous studies have seldom examined the influence of personality, psychological empowerment, and social capital on knowledge sharing and innovation performance. The majority of studies in the literature have explored the following topics: the influence of personality in contexts such as the tourism industry [see Lin and Tang (2018)]; the influence of psychological empowerment in the manufacturing industry (Wang et al., 2019) and education (Khan et al., 2019); the influence of social capital and innovation performance in multinational companies in manufacturing and growing industries (Singh et al., 2021); and the influence of knowledge sharing on the manufacturing (Liao, 2018) and hospitality (Hsu et al., 2021) industries. In summary, the research frameworks relevant to this study have mostly been applied to in studies of the service and manufacturing industries. Although real estate brokerage is part of the service industry as a whole, few studies have applied these research frameworks in that context. In addition to enhancement of professional knowledge, communication, and service are also particularly important in the industry. They are the key determinant of sales. Thus, the findings of this study fill the aforementioned gap in research and are support the future development of the industry.

In political science studies, Mintrom (1973) found that state’s school-choice plans can be promoted by policy entrepreneurs. The diffusion of policy innovation can be horizontal (between states) or vertical (states and local governments). Mintrom and Vergari (1998) suggested that being involved in political networks increases the likelihood of policy entrepreneurs achieving their legislative goals. Lewis et al. (2022) demonstrated that personal attributes significantly influence the willingness of policymakers to engage in policy innovation, and this influence varies in response to changes such as a heightening of risk. Additionally, praise was found to influence their intrinsic and extrinsic motivation to create policies.

Based on statistics from the Ministry of the Interior Real Estate Service Information System (2022) the number of real estate businesses in Kaohsiung City has risen from 513 in July 2021 to 542 to July 2022. A main reason for this is that Taiwan Semiconductor Manufacturing Co., Ltd., (TSMC) is set to construct a new plant in the city, offering vast employment opportunities. Coupled with the anticipatory effects felt within the general public, housing prices in the city have risen (PTS News, 2021) and the real estate brokerage industry is flourishing. For this reason, the focus of this study is the real estate brokerage scene in Kaohsiung. It explores the influence on that industry of four organizational behaviors or psychological dimensions, namely personality, psychological empowerment, social capital, and knowledge sharing. Additionally, with information asymmetry in mind, moral hazard is set as a confounding variable to identify its effect on innovation performance.

A key characteristic of the real estate brokerage industry is that it is highly dependent on humans, from processes such as product development to the provision of professional front-and backend services. Relationships (especially of trust) are essential to the industry’s expansion. The global pandemic over the past 2°years has prolonged working hours at home, and fueled developments in the real estate industry including leveraging online technologies to boost sales (such as enabling house buyers to use virtual reality technology to view houses online). Knowledge sharing is another important tool for achieving innovation performance.

Based on the characteristics of the industry, this study consolidates information on certain human psychological traits (personality and psychological empowerment). It then explores how trust is established through strong interpersonal relationships as a form of social capital, which is then used to achieve knowledge sharing within the organization and promote innovation. We developed a conceptual model to validate the causal relationships between these latent variables. To summarize, the objectives of this study are: (1) To explore the influence of personality and psychological empowerment on social capital; (2) To explore the influence of personality and psychological empowerment on innovation performance; (3) To explore the influence of social capital on knowledge sharing and the influence of knowledge sharing on innovation performance; and (4) To explore how hazard as a confounding variable influences the effect of social capital on knowledge sharing.

## Literature review and hypotheses development

Costa and McCrae (1992) identified five major personality traits, openness, conscientiousness, extroversion, agreeableness, and neuroticism. Adler et al. (2000) argued that social capital emerges in a particularly special relationship (with colleagues and relatives) and is established through interactions with others. The resources or information obtained through exchanges in this mutually beneficial relationship are a form of social capital. According to Allport (1961), personality is a unique model that is characterized by continuity and stability, and determines an individual’s thoughts and behaviors. Due to the fact that differences between individuals are largely shaped by personality, it can be used as a criterion for distinguishing individual differences. Wiggins (1996) pointed out that numerous management thinkers and sociologists have employed personality as a predictor of individual behavior and performance. Through understanding an employee’s personality, supervisors and managers cannot only predict their work performance, but also leverage this information to acquire better talents. Within social studies, social capital refers to the trust, cooperation, and assurance generated through the development of long-term interpersonal relationships. It represents the closeness of interpersonal relationships as well as utilizable resources. Bourdieu and Wacquant (1992) defined social capital as the sum of the resources acquired by an individual or organization through an enduring network of relationships. An enterprise is essentially a community of individuals; and hence the influence of social capital exists in an enterprise’s internal and external networks, links, and relations. In essence, social capital is a form of interpersonal relationship.

Schmitt and Shackelford (2008) wrote that an employee with a personality characterized by agreeableness tends to be perceived as trustworthy and helpful, and easily capable of building positive relationships with other organizational members. In terms of social capital, a highly approachable employee tends to have higher levels of empathy, and is thus more likely to gain the trust of others. Ashton et al. (2002) noted that extroverted individuals are capable of attracting the attention of others and maintaining interpersonal interactions with them, thereby increasing their social capital. Chiang (2004), through performing variation analysis on Taiwanese workers in the manufacturing sector, found that the differences in social capital are significantly influenced by personality. In a study on the owners and managers of real estate stores, You (2012) revealed that personality significantly influences social capital, as well as knowledge-sharing intentions through the mediation effects of social capital. Wang et al. (2021) found the personality of cross-national real estate investors positively affected risk management. In addition to investor personality, the personality of brokers is also important because when investors and brokers develop strong interactions and relations, they generate the mutual trust required to effectively reduce risk-management hazards. Accordingly, personality reflects the level of trust and interactions in social capital. Therefore, we propose the following hypothesis:

**H1:** Personality significantly and positively influences social capital.

Sun (2015) described innovation performance as a process of creating new values and enhancing one’s individual performance by systematizing new concepts or objects into new knowledge. Drucker (1998) pointed out that innovation requires innate talent, independent thinking, and extensive knowledge. However, even if an innovator possesses all these prerequisites, they would fail to achieve their goals without hard work and dedication. Sung and Choi (2009) revealed that extroversion and being open to experience positively influences individuals’ innovation performance. Kwong and Cheung (2003) suggested that, despite personality being an indicator of performance level, due to environmental and behavioral factor-related differences, the predictive validity of innovation performance differs even in the same personality trait. Hasso (2013) noted that personality affects decision-making processes, and is also a predictor of success in innovation. In this study, we suggest that there is an association between the personalities of real estate brokers and their innovation performance. Chai et al. (2020) studied the influence of emotional management on personality and innovative behavior among Taiwanese college students. The results showed that respondents who were more extroverted, agreeable, rigorous, and open performed better in their innovation behaviors. Accordingly, we propose the second hypothesis:

**H2:** Personality significantly and positively influences innovation performance.

Zhang and Bartol (2010); Seibert et al. (2011); Maynard et al. (2012) and concurred that psychological empowerment is a motivational state experienced when one is actively oriented to a work task. It is achieved through the integrative effects of cognition, and enables employees to achieve immediate success, become more invested in their work and work environment, and make that work environment more distinctive. Velthouse (1990) summarized psychological empowerment as a belief in mobilizing one’s capabilities to make and implement decisions. This belief is shaped through the mutual effects of cognitive styles, environmental factors, and expressed behaviors. It is characterized by awareness, confidence, independence, risk tolerance, responsibility, and dedication. According to Fletcher (1992), the greater an employee’s empowerment, the greater their capability for assuming sole responsibility for a work task, and (relatively speaking) the stronger their capacity to express their capabilities in various aspects of social capital. Christens et al. (2011) argued that psychological empowerment is socialized through participation in community empowerment, and interpersonal relations are gradually developed as a result. Wang et al. (2019) explored how different forms of interpersonal conflict and the psychological empowerment of employees directly or indirectly affect knowledge sharing intentions through trust within the workplace. The questionnaire’s 249 respondents were employed at 37 of the top 500 companies in the Taiwanese manufacturing industry. The results revealed that interpersonal relations and tasks conflict directly and significantly affected the employees’ knowledge sharing intentions through psychological empowerment and trust. Psychological empowerment (autonomy, competence, and job influence) achieves stronger interactions through interpersonal trust among the employees, allowing knowledge-sharing spillover.^1^ A wealth of research has demonstrated the positive association between interpersonal relations and psychological empowerment see Zimmerman et al. (1992); Israel et al. (1994); Peterson and Reid (2003). As such, we propose the third hypothesis:

**H3:** Psychological empowerment significantly and positively influences social capital.

According to Sun (2015), innovation performance is the process of new value creation and indicial performance enhancement based on the systematization of new concepts or objects into new knowledge. Spreitzer (1995) argued that the facilitating conditions offered by an organization influences the significance and level of influence perceived by an employee toward their work tasks, as well as their self-efficacy and decision-making skills, thereby enhancing their perception of empowerment and work motivation. Bowen and Lawler (1992) argued that, when employees perceive themselves as having more authority, they have a stronger capability to perform their duties and engage in innovative thinking, thus boosting their confidence and problem-solving skills. In a related study, Kelly et al. (1996) demonstrated that service workers have better service skills when they have a higher level of psychological empowerment, and thus have higher levels of confidence as well as better creative thinking and problem-solving skills. Helmy et al. (2019) administered a questionnaire to 500 employees working at 50 different SMEs in Yogyakarta, Indonesia and showed that the three dimensions of psychological empowerment (meaning, competence, and autonomy) had positive effects on innovative work behaviors. Accordingly, we propose the following:

**H4:** Psychological empowerment significantly and positively influences innovation performance.

Nonaka and Takeuchi (1995) described knowledge sharing as a process in which one exchanges, transfers, or spreads tacit knowledge, making it explicit by interacting with others through a series of communication. Knowledge sharing can only be achieved by interacting and exchanging information with others. Bowen and Lawler (1992) pointed out that reciprocity and trust are of utmost importance toward social capital creation, as both factors determine the level to which one is willing to share one’s resources with others. Social capital strengthens knowledge acquisition. Through leveraging their social capital-based relations, a knowledge sharer acts as a catalyst for transferring or sharing knowledge to a recipient, thereby expediting the process of data collection, increasing the efficiency of knowledge exchange, strengthening the trust between the sharer and recipient, and improving an enterprise’s internal knowledge acquisition skills (Adler and Kwon, 2002). Wu (2020) explored the influence of school knowledge governance on teachers’ knowledge sharing and transfer, with social capital serving as a mediator. The study included 42 junior high schools in four cities in northern Taiwan (Keelung, Taipei, New Taipei, and Taoyuan), from which 700 teachers were administered a questionnaire. The results revealed that teachers’ social capital mediated their knowledge sharing. The abovementioned arguments demonstrate the association between social capital and knowledge sharing. Consequently, we expect that employees with greater levels of social capital have higher knowledge sharing intentions; whereas employees with lower levels of social capital may be unable to exhibit high knowledge sharing intentions due to their comparatively weak level of trust and reciprocity between other organizational members. We thus propose the fifth hypothesis:

**H5:** Social capital significantly and positively influences knowledge sharing.

Anand et al. (2002) suggested that, because knowledge is constantly evolving and developing, most organizations are unable to possess the necessary knowledge within their formal boundaries. Accordingly, this necessitates the acquisition of large amounts of knowledge from external sources by using their connections with external organizations and individuals. Wang and Noe (2010) proposed that, as a knowledge-centered activity, knowledge sharing is the fundamental means for employees to interchange knowledge mutually, contribute to knowledge utilization and innovation, and ultimately the furthering of their organization’s competitive advantage and business performance. Zárraga and Manuel García-Falcón (2003) believed that the multidimensional communication between organizational members facilitates the enhancement of organizational innovation. Furthermore, the process through which organizational members consistently exchange and share knowledge enhances the accrual of an organization’s internal knowledge. Thus, enhancing the process of value creation and exchange can drive innovation and enhance an organization’s innovation performance. We thus propose the following hypothesis:

**H6:** Social capital significantly and positively influences innovation performance.

Biais and Casamatta (1999) and Chen (2016) found that moral hazard entails taking into account the conditions most favorable tor oneself, retaining information, and comparing personal interests. Moral hazard is derived from the information asymmetry between two parties and subsequently leads to opportunism, when the party with the informational advantage fails to fulfill their contractual obligations or begins to work less diligently. This opportunistic behavior increases transaction costs and reduces efficiency. While knowledge sharing is a voluntary behavior, people do not necessarily share information with others due to the innate tendency to preserve one’s own competitive advantage (Weng and Tung, 2017). Almeida and Kogut (1999) proposed the concept of knowledge localization. In an organization, it is difficult to transfer away the information held within a unit which a certain organizational member is less acquainted or familiar with, as people tend to trust those they already know. This implies that individuals with high social capital are strongly expected to share information. Fabella (2013) suggested that, when working together as a team, people work closely with one another because they have common objectives. However, during the information sharing process, team members may choose to conceal information conducive to the team for the sake of their own personal interests, which leads to moral hazard. Anita et al. (2015) concluded that the establishment of interdependent relations in a team can improve its efficiency, but the latent moral hazards may be detrimental to the effectiveness of knowledge sharing between members. Yang et al. (2018) investigated underinvestment and moral hazard using the Home Appreciation Participation Notes. The objective was to reduce the default risk of house buyers and improve the mobility of real estate products without affecting initial housing prices. The results revealed that close interactions between brokers and customers are conducive to the effective development of mutual trust. Additionally, effective communication can be achieved through knowledge sharing and transfer. This allows the buyer and the seller to have a particular understanding of housing prices, thereby facilitating robust cooperation. When brokers choose to conceal or retain information out of personal interest, moral hazard affects their customers’ trust in them, thus hindering the effective sharing and transfer of knowledge. Accordingly, we propose the following:

**H7:** Moral hazard moderates the influence of social capital on knowledge sharing.

## Materials and methods

### Research framework

**FIGURE 1:**
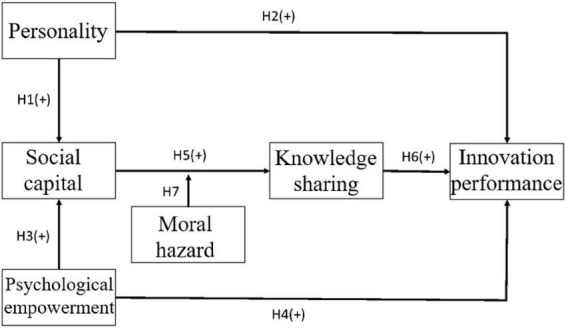
The proposed study framework.

The study framework shown in Figure 1 was analyzed and validated through structural equation modeling (SEM). The framework on knowledge sharing and innovation performance was developed based on Ashton et al. (2002); Knight (2006); Pérez-Luño et al. (2011); and Christens (2012).

### Operational definitions of variables

#### Personality

Costa and McCrae (1992) categorized five dimensions of personality: openness, conscientiousness, extraversion, agreeableness, and emotional stability. These five dimensions do not represent five particular personality types, but are rather a comprehensive summary of the broad characteristics of each dimension. Mayer et al. (2001) synthesized dozens of personality-related studies and pointed out that scholars have reached a general consensus on the five dimensions. A systematic framework on these five dimensions allows individuals to easily distinguish and understand the characteristics of each based on the taxonomy. Given the robust development of Costa and McCrae’s (1992) taxonomy of the five main personality traits, we adopted it as the operational definition of personality.

#### Social capital

Adler et al. (2000) suggested that social capital is a unique and distinct relationship (with colleagues or relatives) that is based on interactions with others. In this mutually beneficial relationship, the resources and information exchanged and received becomes social capital. Nahapiet and Ghoshal (1998) defined social capital as a unique relational resource that consists of interpersonal, group, and social relationship networks. This resource benefits an individual’s personal social and intelligence capital, wealth creation, and achievement of goals. We opted to follow Adler et al. (2000) conceptualization of social capital, and the measures thereof developed by Nahapiet and Ghoshal (1998) and propose an operational definition of social capital that consists of two dimensions: (1) Strong interrelationships, which refers to the strength and frequency of communications between organizational members; and (2) Trust, based on affection and affective combinations.

#### Psychological empowerment

According to Zhang and Bartol (2010); Seibert et al. (2011); and Maynard et al. (2012), psychological empowerment is a motivational state grounded in active orientation to one’s work tasks. It is realized through the integrative effects of cognition and helps employees rapidly achieve success, initiates their work-oriented development, boosts their interest in their work environment, and enables them to create their own work environment. Spreitzer (1995) developed a cognitive model of psychological empowerment, This is primarily assessed through four dimensions: meaning, self-determination, competence, and impact. We have adopted these four dimensions as the operational definition of psychological empowerment.

#### Knowledge sharing

Nonaka and Takeuchi (1995) conceived of knowledge sharing as an interactive process by which one exchanges, and disseminates one’s tacit knowledge, converting it into explicit knowledge through a series of communications. That is, one can only engage in knowledge sharing by interacting with others. Hendriks (1999) classified knowledge sharing into two players and two activities. The two players are the knowledge sharer and the knowledge recipient, while the two activities are externalization and internalization. The former activity is the process of externalizing acquired knowledge through instruction, writing, filing, and database creation. The latter is the process of converting newly-acquired knowledge into one’s own knowledge through such methods as learning by doing and imitation. The level of knowledge sharing with an enterprise is a key determinant of its competitive advantage and influence. This study adopts Nonaka and Takeuchi’s (1995) conception of knowledge sharing, and Hendriks’ (1999) classification, to develop an operational definition of knowledge sharing.

#### Innovation performance

Venkatraman and Ramanujam (1986) state that the ultimate goal of every management and strategy-related activity within an enterprise is to enhance its innovation performance, which Sun (2015) defined as the process of systematizing new conceptions or objects into new knowledge to subsequently create new values with which to improve performance. Schumann et al. (1994) discussed innovation performance along three dimensions: cost reduction, functional enhancement, and pattern changing; we have adopted this as the operational definition of innovation performance.

#### Moral hazard

In both Biais and Casamatta (1999) and Chen (2016) moral hazard entails such actions as considering the most favorable conditions, retaining information, and comparing personal interests. We have adopted this conceptualization as the operational definition of moral hazard.

### Questionnaire design

Our questionnaire consisted of two sections. The first included the participants’ basic information, such as their gender, age, tenure in the real estate industry, income, job position, education level, and business model. The second section covered items pertaining to the variables of personality, social capital, innovation performance, psychological empowerment, knowledge sharing, and moral hazard.

We developed the ten items concerning personality on the basis of studies conducted by Weaven et al. (2009); Wang (2010); and Man (2014). The aforementioned five sub-dimensions (openness, conscientiousness, extraversion, agreeableness, and emotional stability) were each given two items as a sub-dimension. The six items concerning social capital were developed following Nahapiet and Ghoshal (1998) and Adler et al. (2000). There are two sub-dimensions, namely strong interrelationships and trust, for which two and four items were designed for each sub-dimension, respectively. We developed the eight items concerning psychological empowerment on the basis of Spreitzer (1995); Li et al. (2006); and Ma (2004). There are four sub-dimensions—meaning, self-determination, competence, and impact—for which two items were designed for each sub-dimension. The seven items concerning knowledge sharing were developed following Nonaka and Takeuchi (1995); Hendriks (1999); Wu (2011); Chang (2002); and Lin (2013). There are four sub-dimensions—knowledge sharer, knowledge recipient, externalization, and internalization—for which one or two items were designed for each.

The nine items concerning innovation performance were developed following the definitions of Schumann et al. (1994) and Venkatraman and Ramanujam’s (1986), as well as from Sun’s (2015) study. There are three dimensions—cost reduction, performance enhancement, and pattern changing—for which we designed three items for each. The three items concerning innovation performance were developed on the basis of Biais and Casamatta (1999) and Chen (2016). All of the questionnaire items were measured on a five-point Likert scale (1 = strongly disagree; 2 = disagree; 3 = neutral; 4 = agree; 5 = strongly agree) (see Table 1).

**TABLE 1:** Questionnaire items and data sources.

Dimension	Item	Data sources
(1) Personality		
Openness	1. I am a highly imaginative person who likes to think.	Weaven et al., 2009; Wang, 2010; Man, 2014
	2. I often participate in new courses and activities, or accept new conceptions.	
Conscientiousness	1. I am an earnest and responsible person.	
	2. I am a person who goes by the book, and has high self-control and self-discipline.	
Extraversion	1. I often play a proactive role in interpersonal relations and interactions.	
	2. I am able to naturally adapt to new environments.	
Agreeableness	1. I empathize with others’ feelings.	
	2. I am an easy-going person whom others do not feel pressured to be with.	
Emotional stability	1. I am a person who gets anxious or nervous easily.	
	2. I am a person who can easily be influenced by external factors.	
(2) Social capital		
Strong interrelationships	1. When I encounter difficulties and setbacks in my work, I will actively seek assistance from my colleagues.	Nahapiet and Ghoshal, 1998; Adler et al., 2000
	2. My colleagues and I are able to understand each other because we have the same working language and rules.	
Trust	1. I believe that most of my colleagues are trustworthy.	
	2. I believe that my colleagues’ commitments to me are reliable and trustworthy.	
	3. I feel that my company allows its employees to unleash their potential and work together to achieve common goals.	
	4. I share the same expectations and values with my colleagues, which allows us to cooperate to achieve common goals.	
(3) Psychological empowerment		
Meaning	1. To me, the work tasks that I do are extremely meaningful.	Spreitzer, 1995; Ma, 2004; Li et al., 2006
	2. My job is extremely important to me.	
Self-determination	1. I have many opportunities to be independent and make my own decisions in my job.	
	2. I have a high level of autonomy in choosing how I complete my work tasks.	
Competence	1. I possess the skills required to complete my work tasks.	
	2. I am confident that I have the capability to complete every work task correctly.	
Impact	1. I am very confident in my capabilities to complete my work tasks.	
	2. I am highly influential in my department.	
(4) Knowledge sharing		
Knowledge sharer	1. I am very willing to share information with my colleagues when they have questions for me.	Nonaka and Takeuchi, 1995; Hendriks, 1999; Chang, 2002; Wu, 2011; Lin, 2013
	2. I gain a sense of accomplishment when I share knowledge with others.	
Knowledge recipient	1. I am able to more closely address the needs of customers when I receive new information.	
Externalization	1. I listen to the experiences of my colleagues and use them as a reference for moving forward.	
	2. My colleagues share work-related information and knowledge amongst themselves.	
Internalization	1. I observe and learn from other colleagues to strengthen my own knowledge and skills.	
	2. I actively participate in in-service training programs or workshops to acquire new knowledge.	
(5) Innovation performance		
Cost reduction	1. I believe that I am able to provide fast and efficient services to customers.	Venkatraman and Ramanujam, 1986; Schumann et al., 1994; Sun, 2015
	2. I believe that I am able to quickly assimilate new market conceptions.	
	3. I believe that I am able to dedicate myself to serving customers in a timely manner.	
Performance enhancement	1. I believe that the standard of the services that I provide is an important competitive advantage.	
	2. I believe that my experiences in serving customers are more superior than those of my competitors.	
	3. I believe that the standard of my services has attracted continuous usage from customers.	
Pattern changing	1. I believe that my sales performance contributes to the stable growth of my company’s market share.	
	2. I believe that I have a respectable image and reputation in my company.	
	3. I believe that I am more capable of proposing successful strategies ahead of my competitors.	
(6) Moral hazard		
	1. I will prioritize the conditions that benefit me the most.	Biais and Casamatta, 1999; Chen, 2016
	2. I will conceal some information when I converse with others.	
	3. I believe that personal interests outweigh the company’s interests.	

### Sampling and data collection

We employed purposive sampling to survey real estate brokers from seven administrative districts in Kaohsiung City. A questionnaire was administered to the participants in person. The survey period ranged from May 1, 2020 to June 30, 2020. There were 1,000 questionnaires, 571 of which were recovered (111 from Sanmin District, 63 from Lingya District, 119 from Zuoying District, 60 from Cianjhen District, 62 from Sinsing District, 48 from Fongshan District, and 108 from Gushan District). After removing 52 invalid responses, we were left with a final count of 519, thus indicating an effective response rate of 51.9%.

## Descriptive statistics of the sample and reliability and validity analysis

### Descriptive statistics of the sample

The results of the questionnaire survey are provided in Appendix 1. Men and women accounted for 54% and 46% of the sample, respectively (280 males and 239 females). A majority (36.8%, 191 individuals) of the participants were aged between 31 and 40, followed by those under the age of 30 (29.1%, 151 individuals). 48.5% (252 individuals) of the participants were single, while 47.9% (249 individuals) were married. A majority (40.9%, 212 individuals) of the participants had between 1 and 5°years’ experience is the real estate sector, followed by those with 6–10°years’ experience (26.3%, 136 individuals). A majority (27.2%, 140 individuals) of the participants had an income of NT$460,000 to 600,000 (27.2%, 140 individuals), followed by those earning less than NT$300,000 (20.4%, 105 individuals). The majority (86.2%, 449 individuals) of the participants were salespersons, followed by brokers (8%, 41 individuals). A majority of the participants (47.3%, 249 individuals) held a university degree (including 4-and 2-year program degrees), followed by those who held a high school (vocational) diploma (26.5%, 137 individuals). Franchises were the dominant company business model, accounting for approximately 78.8% (412 stores), followed by direct sales operations (21.2%, 107 stores).

### Reliability and validity analysis

#### Reliability analysis

The reliability of a sample is a measure of its accuracy in terms of the consistency and stability of the results. The most common method for testing a sample’s reliability is by using the coefficient developed by L. J. Cronbach, as represented by the following equation 1:


(1) $$\alpha =\frac{K}{K-1}\,\left(1-\frac{\sum S_{i}^{2}}{S^{2}}\right)$$

where *K* represents the total number of questionnaire items, *S*^2^ represents the total variance of the overall score of the questionnaire, and $S_{i}^{2}$ represents the variance in the total score of every item. The reliability of an item as represented by Cronbach’s α should generally range between 0 and 1 (Chiu, 2006). Nunnally (1978) suggested that a Cronbach’s α of 0.70 is a relatively low, but acceptable, borderline value. Cooper and Schindle (2003) suggested that a Cronbach’s α of greater than 0.90, 0.80, and 0.70 indicates high reliability, good reliability, and the minimum threshold for acceptable reliability, respectively. DeVellis (1991) suggested that a Cronbach’s α of between 0.65 and 0.70 is acceptable, between 0.70 and 0.80 being indicative of high reliability, while one greater than 0.80 indicates excellent reliability. All of the Cronbach’s α values in this study were greater than 0.70, thereby indicating high reliability. Therefore, our questionnaire was excellent in terms of stability and consistency (see Table 2).

**TABLE 2:** Reliability analysis of each dimension.

Dimension	Number of items	Cronbach’s α
Personality	10	0.786
Social capital	9	0.858
Psychological empowerment	12	0.882
Knowledge sharing	10	0.899
Innovation performance	12	0.914

#### Validity analysis

We performed a validity analysis in terms of content, convergent, and discriminant validity. Chou (2002) noted that the determination of content validity is a subjective process based on logic, unlike reliability which has many quantitative measurement indicators. A questionnaire with items developed based on logical means, such as theoretical foundations, empirical research, logical reasoning, and expert consensus, tends to be regarded as having considerably high content validity. Our questionnaire centered on the innovative performance of real estate brokers. Previous questionnaires on innovation performance were used as references to design the questionnaire items. We then discussed each item with relevant experts in order to revise the phrasing of each item such that they aligned with the focus on innovation performance. Accordingly, we can consider the content validity of the questionnaire to be reliable.

Anderson and David (1988) and Bagozzi and Yi (1988) suggested that a variable has a good convergent validity when its factor loading is higher than 0.50 and is tested to be statistically significant. Hair et al. (2006) advised that the standardized factor loading of each dimension should exceed 0.5. Furthermore, when the average variance extracted (AVE) of a dimension is high, then the reliabilities of the latent variables are said to be high as well, which indicates good convergent validity. With the exception of emotional stability, the standardized factor loadings of all dimensions exceeded 0.5, thus meeting Hair et al.’s (2006) proposed threshold. All of the factor loadings were also tested to be statistically significant, thus attesting to the good convergent validity of the questionnaire (see Table 3).

**TABLE 3:** Analysis of the scale’s reliability, loading, and variance extracted, and structural modeling estimation results.

Variable	Loading (non-standardized)	Loading (standardized)	Error variance	Reliability of measured variable	Composite reliability (CR)	Average variance extracted (AVE)	Estimated *R*^2^ of structural equation
Personality					0.863	0.580	
Openness	1.000	0.679**	0.230	0.461			
Conscientiousness	1.100**	0.727**	0.213	0.528			
Extraversion	1.167**	0.715**	0.256	0.512			
Agreeableness	1.016**	0.663**	0.259	0.439			
Emotional stability	0.381**	0.235**	0.486	0.055			
Social capital					0.869	0.768	0.741
Strong interrelationships	1.042**	0.707**	0.167	0.499			
Trust	1.000	0.721**	0.141	0.520			
Psychological empowerment					0.918	0.738	
Meaning	0.936**	0.696**	0.239	0.484			
Self-determination	1.031**	0.780**	0.175	0.608			
Competence	1.067**	0.802**	0.162	0.643			
Impact	1.000	0.727**	0.228	0.528			
Knowledge sharing					0.831	0.789	0.754
Knowledge sharer	1.000	0.796**	0.130	0.633			
Knowledge recipient	0.956**	0.701**	0.213	0.491			
Externalization	0.941**	0.756**	0.150	0.571			
Internalization	1.005**	0.798**	0.129	0.637			
Innovation performance					0.939	0.838	0.647
Cost reduction	0.922**	0.773**	0.116	0.597			
Performance enhancement	1.071**	0.852**	0.088	0.726			
Pattern changing	1.000	0.749**	0.159	0.561			

**Denotes p < 0.01.

In terms of discriminant validity, we followed Chin’s (1998) suggestion that the square root of each dimension’s AVE should exceed the correlation coefficients of the other dimensions. This is to say that the square root of each dimension’s AVE must be larger than those in the columns on the left and below in a correlational matrix, so as to ensure a certain level of discrimination between different dimensions. The AVE of each dimension in this study exceeded the correlation coefficients of the other dimensions, indicating that there is a certain level of discriminatory power between the different dimensions in this study (see Table 4).

**TABLE 4:** Correlation matrix of latent variables.

	Personality	Social capital	Psychological empowerment	Knowledge sharing	Innovation performance
Personality	0.762				
Social capital	0.116	0.876			
Psychological empowerment	0.001	0.107	0.859		
Knowledge sharing	0.122	0.161	0.113	0.888	
Innovation performance	0.078	0.122	0.155	0.142	0.915

The diagonal lines represent the square root of the AVE of a dimension.

## Empirical results and discussion

First, we evaluated the theoretical model to confirm whether the overall model fit was acceptable. Once done, we performed linear structural equation modeling.

### Theoretical model

Bagozzi and Yi (1988) proposed three approaches for measuring the fit of a model: overall model fit, preliminary fit criteria, and fit of the model’s internal structure. Each approach is described in the following.

### Preliminary fit criteria

According to Table 3, the factor loadings of the five latent variables were all statistically significant and greater than 0.7. There were also no negative values in the measured error variances. Generally speaking, this study’s model met the preliminary fit criteria. Bagozzi and Yi (1988) proposed five criteria for measuring the fit of a conceptual framework: (1) The measurement errors must not be negative; (2) The measurement errors must be statistically significant; (3) The relative absolute values between the estimated parameters must not be too close to 1; (4) The factor loadings must be neither too low (< 0.5) nor too high (> 0.95); and (5) The standard errors must not be too large. Based on Table 3, the measured factor loadings of the five latent dimensions were all statistically significant and ranged from 0.663 to 0.727, with the exception of emotional stability. In this study, the *R*^2^ of the three structural equations were 0.741, 0.754, and 0.647, respectively. Accordingly, the preliminary fit criteria were generally acceptable.

### Fit of the internal structure of the model

The fit of the internal structure of a model primarily assesses the level of significance of the model’s estimated parameters, as well as the reliability of each indicator and latent variable. Bagozzi and Yi (1988) proposed the following three indicators: (1) Whether the individual item reliability exceeds 0.50 and whether each factor loading is statistically significant. With the exception of emotional stability (0.235), the factor loadings of all other items were larger than 0.50 and statistically significant (see Table 3). (2) Whether the composite reliability (CR) of a latent variable—which reflects the internal consistency of a construct—exceeds 0.60. A higher reliability indicates an indicator having a high consistency. Fornell and Larcker (1981) suggested that an acceptable CR should be greater than 0.60. The CRs in this study ranged from 0.831 to 0.939, thus exceeding the 0.6 acceptance threshold. (3) The AVE represents the explanatory power of a latent variable toward each measured variable. A high AVE suggests that the variable has high reliability and convergence. Fornell and Larcker (1981) suggested that an acceptable AVE should be greater than 0.50. According to Table 3, the AVE of each latent variable ranged from 0.580 to 0.838, meaning that they were all within an acceptable range. In sum, the fit of the internal structure of the questionnaire in this study was deemed acceptable.

### Overall fit of the model

The overall fit of a model is used to assess the fit of the observed data and the overall model. Bagozzi and Yi (1988) stressed that the fit of a structural model cannot be determined through a single indicator or criterion. Instead, the test results of the overall model must be considered. The criteria we used included the chi-square statistic (χ^2^), the normal chi-square statistic (χ^2^/*df*), the goodness of fit index (GFI), the root mean square residual (RMR), root mean square error of approximation (RMSEA), adjusted goodness of fit index (AGFI), normed fit index (NFI), comparative fit index (CFI), parsimonious goodness-of-fit index (PGFI), and parsimonious normed fit index (PNFI). Hair et al. (1998) classified three types of overall model fit measures: absolute fit measures, incremental fit measures, and parsimonious fit measures.

#### Absolute fit measures

Chiu (2006) pointed out that, if the conceptual framework does not fit with the structure of the sample data, then the study’s conceptual model does not have a good fit with the observed data. Since the chi-square statistic is highly sensitive to sample sizes, a large sample size will increase the chi-square statistic, resulting in the rejection of the null hypothesis in favor of the alternative hypothesis. To resolve this issue, it is necessary to consider other fit indices for evaluating the overall model’s fit. Hair et al. (1998) suggested that a fit can be considered acceptable when the GFI, CFI, and NFI are greater than 0.90, and the RMR is smaller than 0.05. Yu (2006) pointed out that an RMSEA smaller than 0.05 indicates good fit, and an RMSEA smaller than 0.08 indicates reasonable fit. As shown in Table 5, the χ^2^ value in this study was 770.961 (*p* = 0.001) and attained a 1% significance level. This suggests that the theoretical model is inconsistent with the distribution of the sample data. A further analysis of the other indicators showed that χ^2^/*df* = 5.976; GFI = 0.870; RMR = 0.090; RMSEA = 0.097, which were all in the proximity of an acceptable fit.

**TABLE 5:** Fit measures of the conceptual framework.

Statistical test		Ideal fit standard	Results
Absolute fit measures	_χ2_ (*p*-value)	770.961	0.001
	_χ2/df_	Smaller than 5	5.976
	*GFI*	Larger than 0.90	0.870
	RMR	The lower the better	0.090
	*RMSEA*	The lower the better, and lower than 0.05 is favorable	0.097
Incremental fit measures	*AGFI*	Larger than 0.90	0.828
	*NFI*	Larger than 0.90	0.862
	*CFI*	Larger than 0.90	0.882
Parsimonious fit measures	*PNFI*	Larger than 0.50	0.727
	*P*G*FI*	Larger than 0.50	0.657

#### Incremental fit measures

Incremental fit measures compare the improvement in the fit of an independent model based on a preset model. As shown in Table 5, the incremental fit measures of this study’s conceptual framework were all in the proximity of an acceptable fit (AGFI = 0.828, NFI = 0.862, CFI = 0.882).

#### Parsimonious fit measures

Parsimonious fit measures refer to adjusted fit measures that determine the goodness of fit that can be obtained by each estimated parameter. As shown in Table 5, the PNFI and PGFI in this study were both greater than 0.50, at 0.727 and 0.657, respectively. Based on the evaluation of all indicators, the overall fit of the theoretical model in this study attained an acceptable level.

### Linear structural equation modeling results and discussion

**FIGURE 2:**
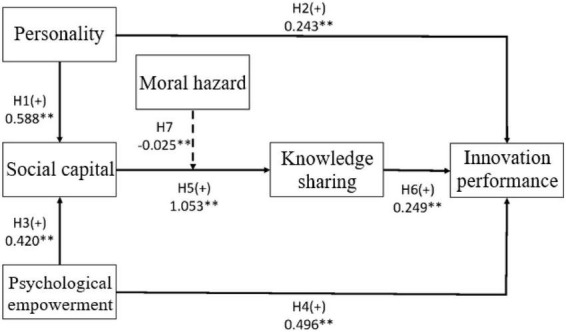
Empirical results of the path analysis. **Denotes *p* < 0.01.

This study employed AMOS-24 statistical software for path analysis. All of the predictors (personality, social capital, psychological empowerment, knowledge sharing, and innovation performance) were placed into the regression model to generate the estimated relation between each variable, as well as to determine whether the hypotheses are supported. The path analysis results are presented in Table 6 and Figure 2. Due to the excellent fit of the overall model, the path model of each hypothesis is supported.

**TABLE 6:** Estimation results of linear structural equation modeling.

Hypothesis	Relationship between variables	Estimated coefficient	Standard error	*t*-ratio	*p*-value
H1	Personality → Social capital	0.588	0.052	11.327	0.001**
H2	Personality → Innovation performance	0.243	0.061	3.947	0.001**
H3	Psychological empowerment → Social capital	0.420	0.039	10.879	0.001**
H4	Psychological empowerment → Innovation performance	0.496	0.054	9.236	0.001**
H5	Social capital → Knowledge sharing	1.053	0.073	14.385	0.001**
H6	Knowledge sharing → Innovation performance	0.249	0.065	3.828	0.001**

**Moderating effects**		**Chi-square (_χ2_)**		**Chi-square difference**	**Hypothesis**
		**original model**	**Competing model**		

H7	Moral hazard _×_ Social capital → Knowledge sharing	900.870	905.635	4.765	Supported

**Denotes p < 0.01.

Based on the empirical results, the estimated coefficient of personality on social capital was 0.588 and attained a 1% significance level. This shows that personality has a significant and positive influence on social capital, i.e., different personalities influence social capital differently. Therefore, H1 is supported. This result validates Halamandaris and Power’s (1997) suggestion that significant differences exist in the influence of different personalities on social capital. The estimated coefficient of personality on innovation performance was 0.243 and attained a 1% significance level, thus supporting H2 by showing that personality has a significant and positive influence on innovation performance. This result is in line with Serarols et al. (2006), who found that the personality traits of entrepreneurs significantly influence the growth of their company, and that the big five personality traits are significantly related to a company’s innovation performance. Hough (2011) noted that distinct classes of personalities serve as predictors of job performance in terms of sales capabilities, leadership skills, and performance.

The estimated coefficient of psychological empowerment on social capital was 0.420 and attained a 1% significance level. This shows that psychological empowerment has a significant and positive influence on social capital, i.e., brokers with strong psychological empowerment exert a high influence on social capital. Therefore, H3 is supported. This result aligns with Christens et al. (2011), who demonstrated that one’s psychological empowerment is socialized through one’s participation in community empowerment, which in turn broadens one’s interpersonal relations. Therefore, the combined effects of personality and psychological empowerment are prerequisites to the factors that influence the social capital of brokers. The estimated coefficient of innovation performance on social capital was 0.496 and attained a 1% significance level. This shows that psychological empowerment has a significant and positive influence on innovation performance, meaning that H4 is thus supported. Lawler (1990) wrote that psychologically-empowered employees are more innovative and productive. Similarly, Bhatnagar (2012) suggested that psychological empowerment influences work engagement, and results in higher innovation and lower turnover. Increasing the psychological empowerment of an employee strongly influences their job engagement and innovation. Knight (2006) agreed that psychological empowerment is closely associated with innovative behaviors. We can thus infer from the empirical results that the innovation performance of real estate brokers is positively influenced by psychological empowerment.

The estimated coefficient of social capital on knowledge sharing was 1.053 and attained a 1% significance level. The results showed that social capital has a significant and positive influence on knowledge sharing, thereby supporting H5. Adler and Kwon (2002) suggested that social capital strengthens knowledge acquisition. By leveraging their social capital-based relations, a knowledge sharer acts as a catalyst for transferring or sharing knowledge to a recipient, thereby expediting the process of data collection, increasing the efficiency of knowledge exchange, strengthening the trust between the sender and the recipient, and improving an enterprise’s internal knowledge acquisition skills. Therefore, the effects of social capital must be considered when examining the factors influencing brokers’ knowledge sharing. Our empirical results indicate that brokers’ willingness to share knowledge is influenced by their social capital.

The estimated coefficient of knowledge sharing on innovation performance was 0.249 and attained a 1% significance level, thus demonstrating knowledge sharing’s significant and positive influence on innovation performance. Accordingly, H6 is supported. Joseph (1998) suggested that knowledge sharing is a consistent and important component of knowledge management. An organization is unable to collect sufficient knowledge for management purposes when its members are unwilling to share knowledge, nor can it evoke creative ideas through knowledge sharing between members. Fan and Ku (2010) showed that members who are willing to share knowledge positively influence the performance of their organization, are conducive to satisfying the needs of customers, and appreciate the value and learning from other members. Additionally, Calantonea et al. (2002) revealed that organizations that establish learning-oriented environments can catalyze highly active processes of knowledge interchange, and thereby improve their innovation capacity and performance. Knowledge sharing is a necessity for the innovative outcomes of a company, because innovation essentially combines existing methods with new processes through external knowledge systems. Both Chesbrough (2003) and Huizingh (2011) have demonstrated the positive and significant influence of knowledge sharing on brokers’ innovation performance.

Regarding moderating effects, we classified moral hazard into two levels: high and low. As shown in Figure 2, the χ^2^ of the original model (also the baseline model) was 900.870 (df = 258, *p* < 0.001). The original model represents there being no invariance assumptions across samples, that is, the high and low moral hazard groups are a combination of structurally similar models that are independent of, and unassociated with, one another. The χ^2^ of the competing model was 905.635 (df = 259, *p* < 0.001). We obtained the competing model by adding constraints into the original model, which is to say by assuming that moral hazard was set as two (high and low) groups with the same path coefficient. The difference (*Δχ*^2^) between the chi-square statistic of the original and competing models was 4.765, and statistically significant. Since the difference between the two models lay in the addition of constraints, the significant differences in the chi-square statistics of both rejected the constraints. Therefore, the estimated paths of the high and low moral hazard groups in social capital and knowledge sharing differed (0.836 and 0.861, respectively; see Table 7). Furthermore, the addition of moral hazard as a moderating variable significantly dampened the influence of social capital on knowledge sharing in the high moral hazard group. This finding suggests that social capital has a positive influence on knowledge sharing. However, given that humans have an innate tendency to conceal advantageous information, one may choose to not share information with others for the sake of one’s own competitive advantage. In the real estate market, which is marked by imperfect information, the information asymmetry between two parties is conducive to opportunism. As the party with informational advantage fails to fulfill their contractual obligations or works less rigorously, moral hazard will diminish the effect of social capital and knowledge sharing. A summary of the outcomes of the hypothesis testing is provided in Table 8.

**TABLE 7:** Path analysis of the moderating variable.

Path	Confounding variable	Influenced by moral hazard	Uninfluenced by moral hazard	Difference between coefficients
Social capital → Knowledge sharing	Moral hazard	0.836	0.861	−0.025**

**Denotes p < 0.01.

**TABLE 8:** Summary of the outcomes of hypothesis testing.

	Hypothesis	Outcome
H1	Personality significantly and positively influences social capital.	Supported
H2	Personality significantly and positively influences innovation performance.	Supported
H3	Psychological empowerment significantly and positively influences social capital.	Supported
H4	Psychological empowerment significantly and positively influences innovation performance.	Supported
H5	Social capital significantly and positively influences knowledge sharing.	Supported
H6	Social capital significantly and positively influences innovation performance.	Supported
H7	The influence of social capital on knowledge sharing is moderated by moral hazard.	Supported

## Conclusions and recommendations

This study examined the influence of personality, social capital, psychological empowerment, and knowledge sharing on the innovative performance of real estate brokers. Our conceptual framework was supported by the goodness-of-fit test results of the structural equation model.

Theoretically, our findings supported all seven hypotheses: personality and psychological empowerment positively affect social capital and innovation performance; social capital positively affects knowledge sharing; knowledge sharing positively affects innovation performance; and moral hazard confounds the influence of social capital on knowledge sharing.

In practice, even though real estate is part of the service industry, its most distinctive feature is that the product is one not regularly purchased. Real estate is characterized by high price and is affected by locational, environmental, transportation, and economic factors. While it is relatively easy to become a real estate broker, salary, service, and work hours affect the mobility of brokers, whose tasks cover connecting with people, objects, services, and professional knowledge. Thus, personality (being open, hard-working, outgoing, agreeable, and emotionally sensitive) and psychological empowerment (work meaning, autonomy, competence, and job influence) are factors that are important to real estate brokers. Social capital (interaction intimacy and trust) reinforce the relationship between personality and psychological empowerment. With the rise of information technology, knowledge has become the greatest asset. Thus, establishing trust between the organization’s members, provoking their willingness to share knowledge, and achieving healthy competition is conducive to teamwork. Additionally, innovativeness is an indispensable trait for enhancing an individual’s professionalism. The information recipient and sharer can enhance their own competence and competitiveness based on the diverse experiences of their colleagues or through in-service education programs. Nevertheless, moral hazards are very likely to arise in industries that serve people. Real estate brokers often put their own interests first when they encounter competition from their colleagues or rival companies. This puts them at risk of a situation of moral hazard that terminates relationships and knowledge sharing. Our findings are consistent with what is seen in practice practical situations. Therefore, personality, psychological empowerment, and social capital generate knowledge spillover through mutual trust and cooperation, and are conducive to enhancing innovation performance, and industry sustainability.

In this study, we identified a relationship between social responsibility and employee mobility. Future studies could undertake an in-depth exploration of these two variables, so as to enhance the level of innovation in real estate brokerage.

## Data availability statement

Publicly available datasets were analyzed in this study. This data can be found here: C-CL (lcc@mail.nptu.edu.tw).

## Ethics statement

Ethical review and approval was not required for the study on human participants in accordance with the local legislation and institutional requirements. Written informed consent from the patients/participants or patients/participants legal guardian/next of kin was not required to participate in this study in accordance with the national legislation and the institutional requirements.

## Author contributions

H-CC: topic conception, research method, and revision. C-CL: research methods and writing and revision. W-CY: writing and revision. Y-LC: data collection and questionnaire survey. All authors contributed to the article and approved the submitted version.

## Footnotes

1 Community empowerment is a process in which governments grant local community residents more participatory power and influence in public services-related decision-making. The policy orientation emphasizes the role of autonomous organizations and community departments in social policy systems so as to improve the complex interactions and relations between the government and civil society.

## Appendix

**Appendix 1:** Descriptive statistics of the sample.

Variable		Number of participants	Effective percentage
Gender	Male	280	54%
	Female	239	46%
Age	Less than 30°years of age	151	29.1%
	31–40	191	36.8%
	41–50	98	18.9%
	51–60	77	14.8%
	60°years and above	2	0.3%
Marital status	Single	252	48.5%
	Married	249	47.9%
	Others	18	3.6%
Tenure	Less than 1°year	131	25.3%
	1–5°years	212	40.9%
	6–10°years	136	26.3%
	11–15°years	26	5.0%
	16–20°years	8	1.3%
	21–25°years	3	0.6%
	26°years and above	3	0.6%
Annual income	Less than NT$300,000	105	20.4%
	NT$310,000 to 450,000	77	14.9%
	NT$460,000 to 600,000	140	27.2%
	NT$610,000 to 750,000	60	11.5%
	NT$760,000 to 900,000	41	7.8%
	NT$910,000 to 1.05 million	34	6.5%
	NT$1.06 to 1.2 million	18	3.3%
	NT$1.21 to 1.35 million	5	0.8%
	Over NT$1.36 million	39	7.6%
Position	Salesperson	449	86.2%
	Broker	41	8.0%
	Branch manager	29	5.7%
Education level	High school (vocational) education or below	137	26.5%
	Specialized education	126	24.4%
	University (including 4-and 2-year programs	247	47.3%
	Master’s degrees and above	9	1.7%
Business model	Direct sales	107	21.2%
	Franchise branch	412	78.8%
